# Global burden of hepatitis B attributable to modifiable risk factors from 1990 to 2019: a growing contribution and its association with socioeconomic status

**DOI:** 10.1186/s12992-023-00922-z

**Published:** 2023-03-31

**Authors:** Minmin Wang, Liang Yan, Jia Wang, Yinzi Jin, Zhi-Jie Zheng

**Affiliations:** 1 Department of Global Health, School of Public Health, Peking University, 38 Xue Yuan Road, Haidian District, Beijing, 100191 China; 2 Institute for Global Health and Development, Peking University, Beijing, China; 3 Key Laboratory of Carcinogenesis and Translational Research (Ministry of Education/Beijing), Department of Hepato-Pancreato-Biliary Surgery, Peking University Cancer Hospital and Institute, Beijing, China; 4 Key Laboratory of Carcinogenesis and Translational Research (Ministry of Education/Beijing), Peking University Cancer Hospital & Institute, Beijing, China

**Keywords:** Hepatitis B, Alcohol use, Tobacco, Body-mass index, Lifestyle intervention

## Abstract

**Background:**

Hepatitis B is a global public health concern, and modifiable risk factors can accelerate progression of this disease. The burden of hepatitis B attributable to modifiable risk factors has not been well evaluated. We aimed to estimate the disease burden of hepatitis B attributable to tobacco, alcohol use, and a high body mass index (BMI) to guide lifestyle interventions in the management of patients with hepatitis B virus (HBV) infection.

**Results:**

In 2019, 33.73% of hepatitis B age-standardized deaths and 34.52% of disability-adjusted life-years (DALYs) were attributable to tobacco, alcohol use, and a high BMI. The proportion showed an increasing trend that 28.23% of deaths and 27.56% of DALYs were attributable to the three modifiable risk factors in 1990. The hepatitis B burden attributable to modifiable risk factors was disparate across regions and countries. Countries with a low socioeconomic status have a high burden of hepatitis B owing to modifiable risk factors. Countries with a high-level sociodemographic index also had an increasing burden of hepatitis B attributable to a high BMI.

**Conclusions:**

Lifestyle interventions are warranted in hepatitis prevention strategies and plans of action. Countries with low and middle socioeconomic development should be prioritized, and countries with high socioeconomic development should be aware of the novel challenge of a high BMI-related disease burden.

**Supplementary Information:**

The online version contains supplementary material available at 10.1186/s12992-023-00922-z.

## Background

Hepatitis B is one of the most concerning public health issues, and modifiable risk factors could accelerate progression of this disease. Hepatitis B virus (HBV) is a viral infection that attacks the liver and can cause acute and chronic disease. In 2019, the global prevalence of chronic HBV infection (CHB) was 4.1%, representing 316 million people living with HBV [1–3]. Chronic viral hepatitis can progress to life-threatening complications, such as cirrhosis and hepatocellular carcinoma. Depending on the life expectancy, more than 20% of individuals with chronic infection develop end-stage diseases [4, 5], and this resulted in 555,000 global deaths in 2019 [3].

Alcohol use, tobacco, and a high body mass index (BMI) could complicate CHB and accelerate progression towards end-stage liver disease. A study on a representative general population in northern Italy showed that alcohol intake independently predicted death of liver cancer in patients with HBV infection [6]. A narrative review summarized this phenomenon and proposed the possible mechanism that alcohol use decreases spontaneous clearance of HBV and increases the risk of liver disease progression in a dose-response relationship [7]. A population-based study in China reported super-additive and super-multiplicative interactions between HBV infection and tobacco smoking [8]. This interaction was similarly reported in a pooled analysis involving nine publications, which suggested that tobacco smoking increases the risk of liver cancer in the presence of HBV infection [9]. Metabolic dysfunction is an essential contributory cause of liver disease in people with CHB. A prospective, longitudinal cohort study suggested that participants with overweight and obesity had an elevated risk of developing liver cancer and causing liver-related death in a 14.7-year follow-up [10]. Epidemiological evidence has emphasized the role of modifiable risk factors as cofactors in morbidity and mortality related to HBV infection.

Global initiatives have been carried out to control and eliminate viral hepatitis, but lifestyle interventions are undervalued. In May 2016, the World Health Organization (WHO) endorsed the *Global health sector strategy on viral hepatitis 2016–2021* [11]. This strategy proposes the elimination of viral hepatitis as a public health threat and calls for the elimination of viral hepatitis by 2030. This strategy sets the interim targets of reducing new infections by 30% and mortality by 10% in 2020, and the final target of a 95% reduction in new cases and a 65% reduction in deaths by 2030. Priority actions include hepatitis B vaccination, prevention of vertical transmission, testing, diagnosis, and HBV treatment according to the updated WHO guidelines. Although the WHO guidelines recommend conducting alcohol reduction in treatment of people with chronic hepatitis B infection [12], the intervention is not well adopted in country-level action plans [13, 14]. Therefore, estimating the global burden and trends of hepatitis B attributable to modifiable risk factors (alcohol use, tobacco, and a high BMI) at global, regional, and national levels could emphasize the importance of lifestyle intervention to achieve the global target of viral hepatitis elimination by 2030.

In this study, we retrieved data of deaths and disability-adjusted life-years (DALYs) of hepatitis B attributable to alcohol use, tobacco, and a high BMI in 204 countries and territories between 1990 and 2019. We aimed to describe the disease burden and trend by geographic and sociodemographic groups. Our results could be helpful in determining whether there is an emergent need to conduct lifestyle interventions in HBV infection management.

## Methods

### Data sources

The data of disease burden related to hepatitis B were obtained from the Global Burden of Diseases, Injuries, and Risk Factors Study (GBD) 2019 dataset using the Global Health Data Exchange (https://ghdx.healthdata.org). The Institute for Health Metrics and Evaluation initiated the series of GBD studies in 2002. This institute reported the global, regional, and country-level disease burden estimation attributable to metabolic, environmental, occupational, and behavioral risk factors based on a comparative risk assessment framework [15, 16]. In this study, we extracted the deaths and DALYs of hepatitis B attributable to alcohol use, tobacco, and a high BMI at the global level and regionally for 204 countries and territories from 1990 to 2019.

### Definition and estimation framework

The disease burden related to hepatitis B was defined as cirrhosis due to hepatitis B, liver cancer due to hepatitis B, and acute hepatitis B [17]. The term “hepatitis B” refers to the aggregation of these three diseases in this article. Alcohol use was estimated as grams/day of pure alcohol consumed among current drinkers, with consideration of the proportion of individuals who consumed at least one alcoholic beverage in a 12-month period, alcohol consumption, and alcohol liters per capita stock. Tobacco use was defined as current smoking of any tobacco product and former smoking of any tobacco product (smoked tobacco, cigarettes, hookah, and other smoked tobacco products, such as cigars or pipes). A high BMI in adults was defined as a BMI greater than 20 to 25 kg/m^2^ for adults aged older than 20 years as consistent with the GBD 2019 estimates [18]. The term “modifiable risk factors” refers to tobacco, alcohol use, and a high BMI in this article.

Deaths and DALYs of hepatitis B attributable to tobacco, alcohol use, and a high BMI were estimated using the GBD 2019 dataset based on a modeling strategy. Briefly, the relative risks of risk-outcome pairs were summarized based on systematic reviews of published evidence [18]. The exposure of risk factors by age-sex-location-year was then formulated using population survey data with individual-level information and applying spatiotemporal Gaussian process regression or Bayesian statistical models. Finally, the attributable disease burden was computed for the defined risk-outcome pairs. The detailed methodologies and input data are reported elsewhere [18].

The socio-demographic index (SDI) was extracted from the GBD 2019 dataset for representation of the country-level socioeconomic development status [19]. The SDI is a geometric average of 0 to 1 in each country or region. The SDI is obtained by combining the total fertility rate of women younger than 25 years, the education level of people aged 15 years and older, and the lag of per capita income distribution [17]. Countries and regions were divided into five levels according to the SDI as follows: high (> 0.81), high-middle (0.70–0.81), middle (0.61–0.69), low-middle (0.46–0.60), and low (< 0.46).

### Statistical analysis

The numbers of death and DALYs with 95% uncertainty intervals (UIs) were estimated to quantify the burden of hepatitis B attributable to tobacco, alcohol use, and a high BMI globally and regionally in 204 countries and territories from 1990 to 2019. The age-standardized rate (per 100,000 population) was calculated based on the GBD reference population [20]. The average annual percentage change (AAPC) and 95% confidential interval (CI) were calculated with the Joinpoint model (Joinpoint Regression program, http://surveillance.cancer.gov/joinpoint) to evaluate the time trend from 1990 to 2019. The disease burden and time trend were separately estimated and compared by regions, SDI quintiles, and countries. Statistical analysis was performed using Stata (Version 14.0; Stata Corp LLC, TX, USA). All tests were two-sided, and *P* values < 0.05 were considered statistically significant.

## Results

### Burden of hepatitis B attributable to modifiable risk factors

The global, regional, and country-level disease burdens of hepatitis B attributable to tobacco, alcohol use, and a high BMI were estimated (Supplemental Table 1). In 2019, 38,068.53 (95% UI: 20,928.86, 56,370.11) deaths and 1,069,239.47 (95% UI: 563,985.53, 1,627,720.20) DALYs of hepatitis B were attributable to tobacco. Additionally, 125,284.25 (95% UI: 72,232.01, 183,697.10) deaths and 4,468,355.30 (95% UI: 2,696,885.00, 6,440,347.57) DALYs of hepatitis B were attributable to alcohol use. Furthermore, 24,073.53 (95% UI: 8741.33, 48,325.58) deaths and 362,256.65 (95% UI: 88,411.08, 895,554.92) DALYs of hepatitis B were attributable to a high BMI. From 1990 to 2019, the disease burden of hepatitis B attributable to a high BMI significantly increased. The age-standardized rate of hepatitis B attributable to a high BMI increased from 0.25 (95% UI: 0.06, 0.62) to 0.29 (95% UI: 0.10, 0.58) per 100,000 population for deaths, and from 8.23 (95% UI: 2.02, 20.29) to 8.86 (95% UI: 3.15, 17.47) per 100,000 population for DALYs, with an AAPC of 0.43 (95% CI: 0.24, 0.62) and 0.24 (95% CI: 0.05, 0.43), respectively.

**Fig. 1:**
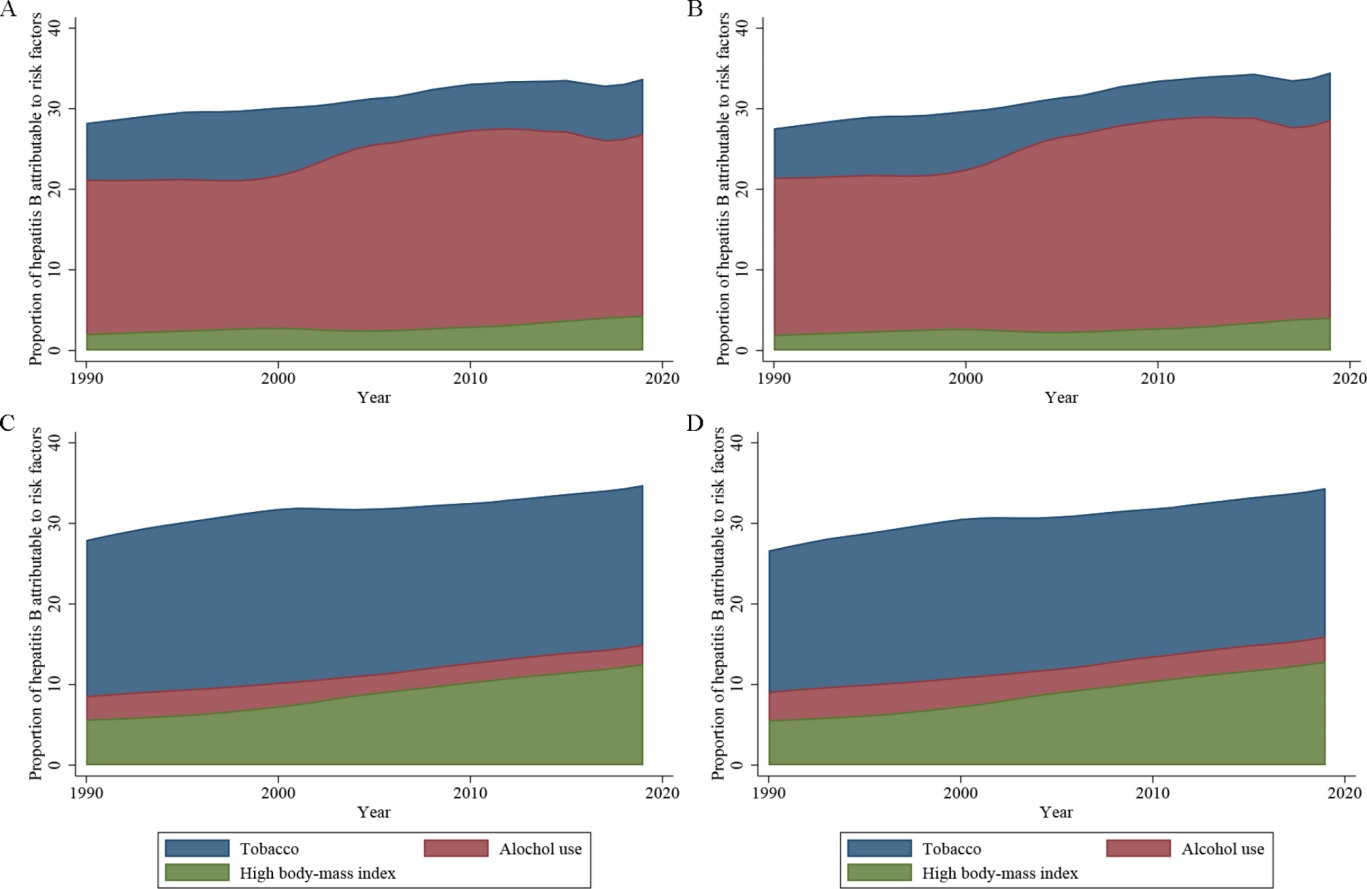
Proportion of burden of hepatitis B attributable to modifiable risk factors, both sexes combined, from 1990 to 2019. (A) Death of total burden related to hepatitis B; (B) DALY of total burden related to hepatitis B; (C) Death of liver cancer burden related to hepatitis B; (D) DALY of liver cancer burden related to hepatitis B. DALY, disability-adjusted life-years

In 2019, 33.73% of hepatitis B age-standardized deaths and 34.52% of DALYs were attributable to tobacco, alcohol use, and a high BMI, and this fraction was increased from 1990, which were 28.23% for deaths and 27.56% for DALYs (Fig. 1). A similar pattern was observed for liver cancer due to hepatitis B in which approximately one-third of deaths and DALYs were attributable to modifiable lifestyle risk factors. The constitution of the three lifestyle factors was different between the total burden related to hepatitis B and liver cancer due to hepatitis B (Supplemental Fig. 1). Alcohol use accounted for a large proportion of the total burden of hepatitis B, whereas tobacco was the main risk factor for liver cancer due to hepatitis B among the three modifiable risk factors.

### Hepatitis B burden attributable to modifiable risk factors by regions and countries

The hepatitis B burden attributable to modifiable risk factors was disparate across the 21 GBD regions (Supplemental Table 1). In 2019, the highest age-standardized rate of tobacco-related hepatitis B was identified in East Asia, with 1.22 (95% UI: 0.66, 1.83) deaths and 34.69 (95% UI: 17.64, 53.33) DALYs per 100,000 population. For the disease burden related to alcohol use, western Sub-Saharan Africa showed the highest burden of 6.72 (95% UI: 3.57, 10.23) deaths and 201.43 (95% UI: 111.81, 306.47) DALYs per 100,000 population. East Asia also had the highest burden of hepatitis B attributable to a high BMI, with 0.63 (95% UI: 0.20, 1.35) deaths and 20.16 (95% UI: 6.37, 43.08) DALYs per 100,000 population in 2019. From 1990 to 2019, the age-standardized death and DALY rates of hepatitis B attributable to tobacco significantly increased in Central Asia, eastern Europe, Australasia, western Europe, southern Latin America, and high-income North America, which was opposite to the contemporary global trend. An increasing trend of hepatitis B attributable to alcohol use was observed in southeast Asia, eastern Europe, and South Asia. Fifteen of 21 regions showed a significant escalating trend for the burden of hepatitis B attributable to a high BMI.

**Fig. 2:**
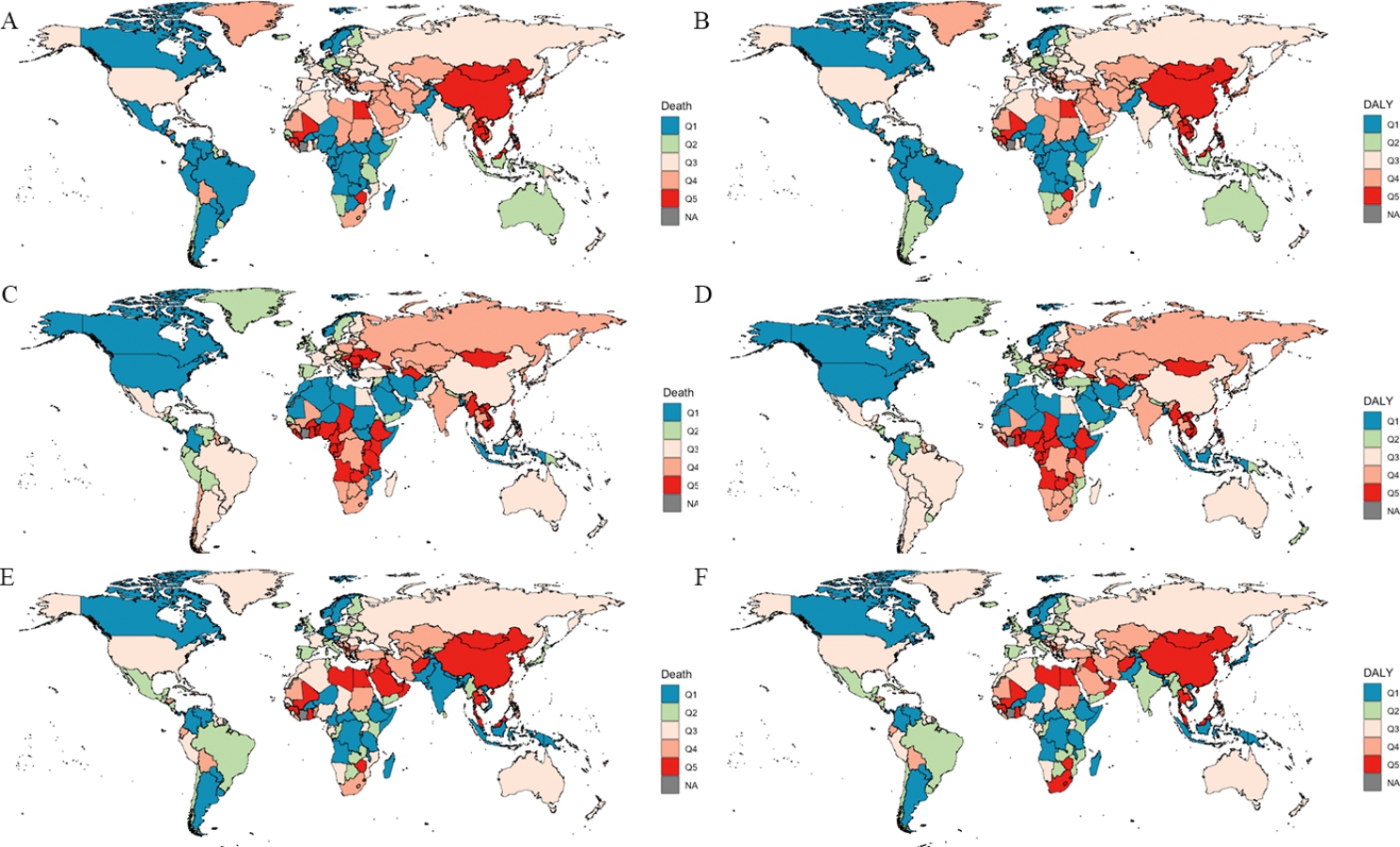
Global map of age-standardized death or DALY rate quintiles for risk-attributable HBV related burden, both sexes combined, in 2019. The age-standardized rate attributable to each modifiable risk factors was categorized in five quintiles and displayed in “Q1” to “Q5”. (A) Tobacco attributable death rate; (B) Tobacco attributable DALY rate; (C) Alcohol use attributable death rate; (D) Alcohol use attributable DALY rate; (E) High BMI attributable death rate; (F) High BMI attributable DALY rate. BMI, body mass index; DALY, disability-adjusted life-years; NA, not available

The distribution of the disease burden in 204 countries and territories is shown in Fig. 2. In 2019, Mongolia had the highest burden of tobacco and high BMI-related hepatitis B, in which the age-standardized death and DALY rates attributable to tobacco were 4.33 (95% UI: 1.68, 7.77) and 115.52 (95% UI: 40.17, 211.397), and those attributable to a high BMI were 4.82 (95% UI: 1.81, 9.33) and 130.01 (95% UI: 49.59, 270.01) per 100,000 population. The burden of alcohol-related hepatitis B reached the highest level in Sao Tome and Principe with 10.37 (95% UI: 5.10, 17.70) deaths and 306.78 (95% UI: 158.42, 503.88) DALYs per 100,000 population.

Age-specific rates of risk factors related to the burden of hepatitis B showed an inverse U shape and peaked at approximately 70 years for deaths and at 60 years for DALYs (Supplemental Fig. 2).

### Hepatitis B burden attributable to modifiable risk factors by SDI quintiles

**Fig. 3:**
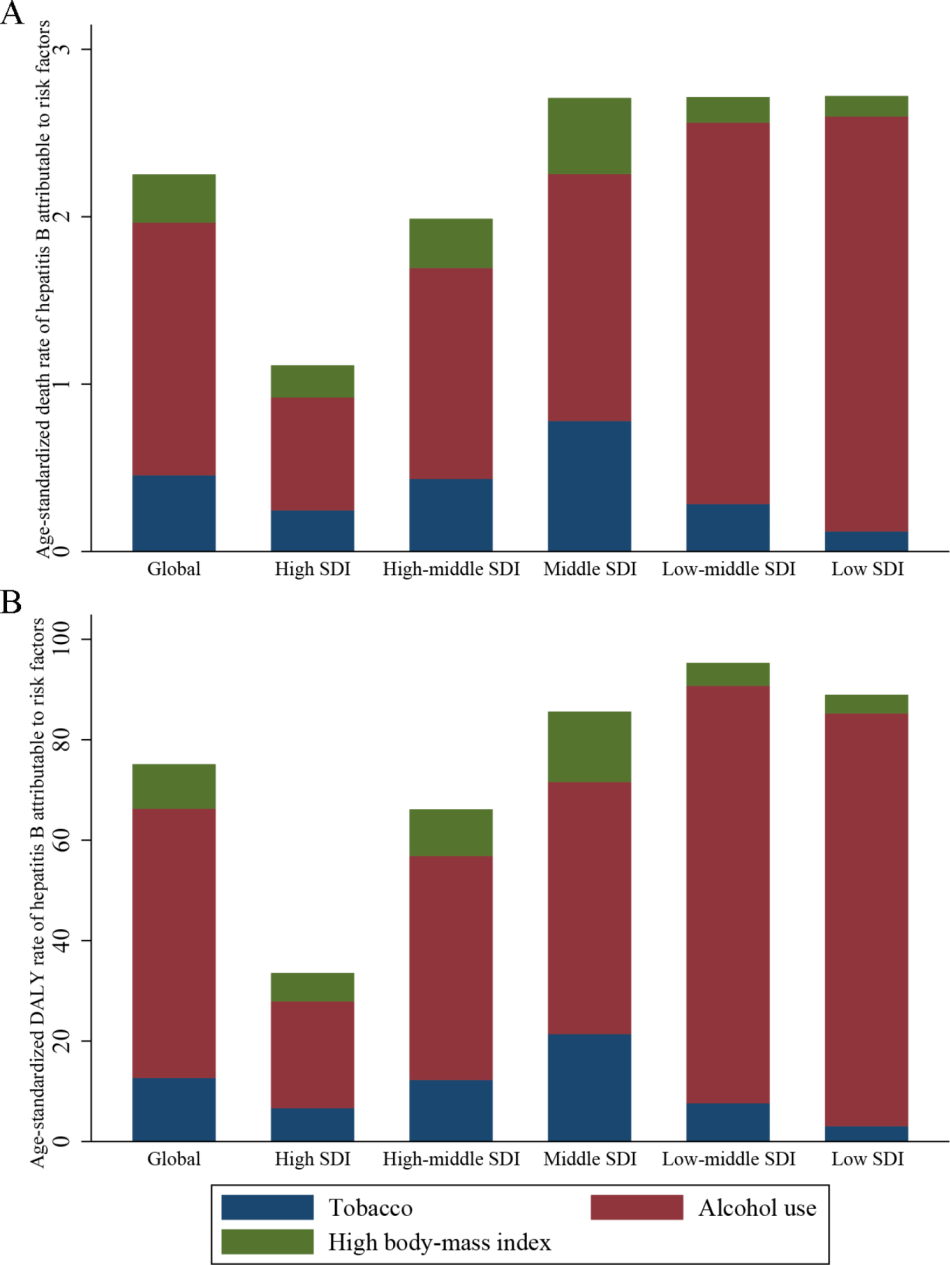
The age-standardized death or DALY rate for risk-attributable HBV related burden by SDI quintiles, both sexes combined, in 2019. (A) Death rate; (B) DALY rate. BMI, body mass index; DALY, disability-adjusted life-years; SDI, socio-demographic index

Countries with a low socioeconomic status have a high burden of hepatitis B owing to modifiable risk factors. The pattern was different across the three risk factors. In 2019, the highest age-standardized rates of hepatitis B attributable to tobacco were observed in countries in the middle SDI quintile, with 0.78 (95% UI: 0.43, 1.15) deaths and 21.24 (95% UI: 11.19, 32.57) DALYs per 100,000 population (Supplemental Tables 2, Fig. 3). However, countries in the low SDI quintile had the lowest burden of 0.12 (95% UI: 0.05, 0.21) deaths and 3.06 (95% UI: 1.02, 5.44) DALYs per 100,000 population. A similar pattern was observed for the hepatitis B burden attributable to a high BMI. The distribution pattern was different for the alcohol-related burden where countries with low and low-middle SDI quintiles had the highest burden, with 2.48 (95% UI: 1.27, 3.97) deaths and 83.13 (95% UI: 43.56, 129.09) DALYs per 100,000 population. Countries with a high SDI quintile had the lowest burden related to alcohol use, with 0.67 (95% UI: 0.41, 0.95) deaths and 21.24 (95% UI: 13.14, 29.67) DALYs per 100,000 population.

**Fig. 4:**
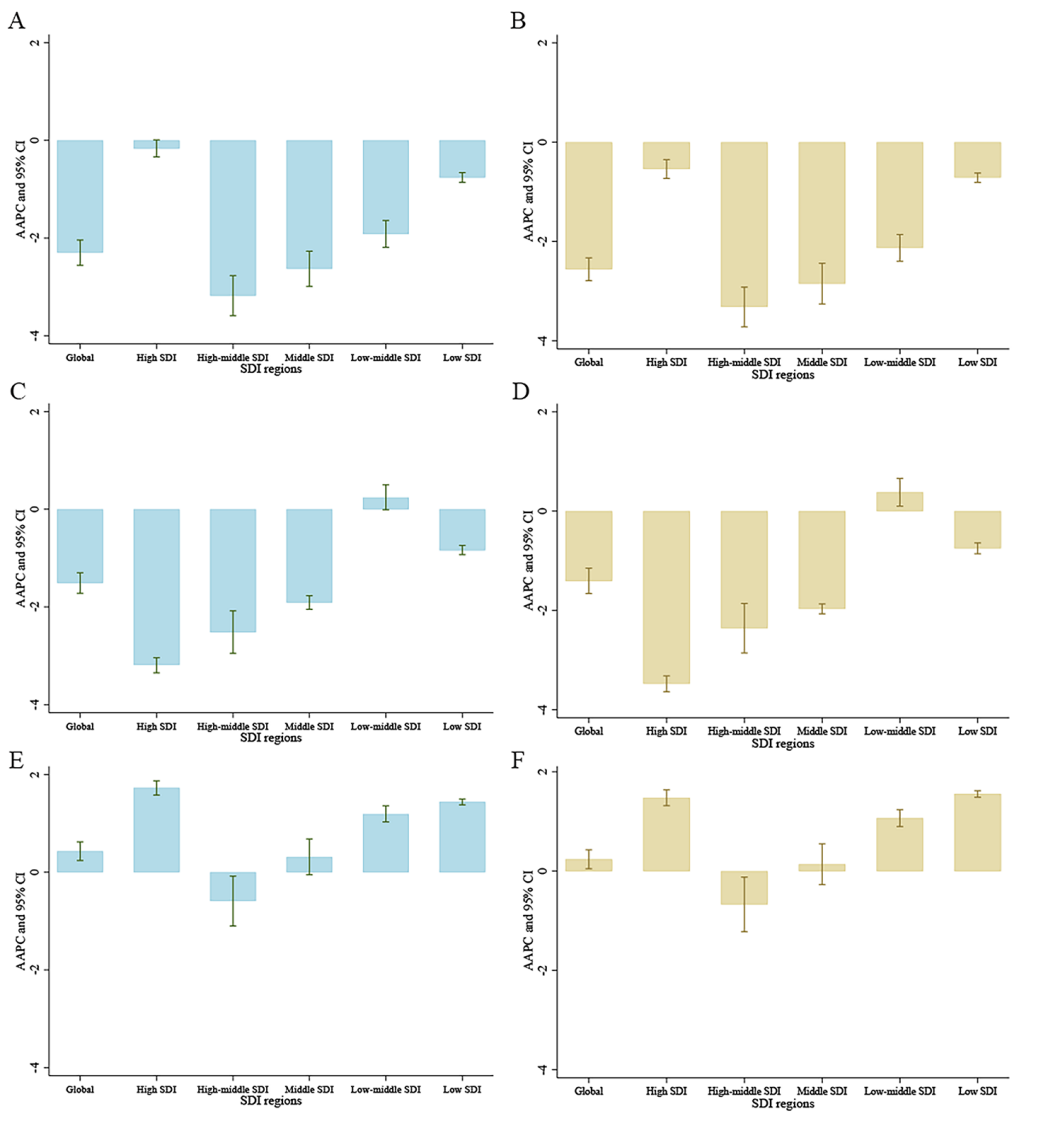
The average annual percentage change of age-standardized death or DALY rate for risk-attributable HBV related burden by SDI quintiles, both sexes combined, from 1990 to 2019. (A) Tobacco attributable death rate; (B) Tobacco attributable DALY rate; (C) Alcohol use attributable death rate; (D) Alcohol use attributable DALY rate; (E) High BMI attributable death rate; (F) High BMI attributable DALY rate. AAPC, average annual percentage change; BMI, body mass index; CI, confidence interval; DALY, disability-adjusted life-years; SDI, socio-demographic index

Figure 4 shows the AAPC in the age-standardized death or DALY rate for risk-attributable HBV burden by SDI quintiles. An increasing trend was observed in the burden of hepatitis B attributable to a high BMI at the global level, and in countries with high, low-middle, and low SDI quintiles. The age-standardized DALY rate of hepatitis B caused by alcohol use was only significantly increased in countries with a low-middle SDI. All regions showed a declining trend of tobacco-related hepatitis B burden from 1990 to 2019.

## Discussion

Hepatitis B is a global public health concern, and appropriate interventions are fundamental to achieve the global target of eliminating hepatitis by 2030. HBV vaccines and antiviral treatment are vital strategies, whereas the role of lifestyle interventions is undervalued in relief of HBV related disease burden [13, 21]. In this study, we evaluated the disease burden of hepatitis B attributable to modifiable risk factors globally and regionally in 204 countries and territories between 1990 and 2019. We found that one-third of the total disease burden was attributable to tobacco, alcohol use, and a high BMI, and this proportion slowly increased from 1990 to 2019. Among the three modifiable risk factors, the age-standardized death or DALY rate of hepatitis B attributable to a high BMI showed a significant increasing trend. Although the burden of hepatitis B attributable to tobacco and alcohol use was declining, regional disparities in the disease burden and temporal trends were different among countries with different SDIs. The estimation of the preventable disease burden has important policy implications in monitoring and managing patients with HBV infection.

Lifestyle interventions are important for preventing disease from infectious causes. The natural history of hepatitis B is dynamic and complex and progresses nonlinearly through several recognizable phases, and the corresponding intervention packages include prevention, diagnosis, treatment, and chronic care. Traditional strategies emphasize viral hepatitis vaccines and antiviral therapy for CHB, but lifestyle interventions have not been the focus of attention. An example of this lack of focus is that the Chinese guidelines for the prevention and treatment of chronic hepatitis B (version 2019) [14] state that harmful alcohol consumption and obesity accelerate the progression from CHB to cirrhosis, and clinical practice guidelines promoted by the European Association for the Study of the Liver [13] also highlighted unfavorable modifiable risk factors in the host that affect disease progression, however, no intervention targeting harmful lifestyle factors was ever mentioned in these guidelines. In this study, we found that > 30% of the total burden of hepatitis B was attributable to tobacco, alcohol use, and a high BMI, and this proportion gradually increased from 1990 to 2019. This growing contribution suggests an urgent need for adding lifestyle interventions to viral hepatitis prevention. A possible intervention is determining alcohol intake for all patients with CHB followed by the offer of alcohol reduction as recommended in the recently published *Global health sector strategies on, respectively, HIV, viral hepatitis and sexually transmitted infections for the period 2022–2030* [22]. Apart from alcohol use, we also found that a large proportion of the hepatitis B-related liver cancer burden was attributable to tobacco use. Therefore, the role of tobacco in liver cancer carcinogenesis should be investigated and transformed into a plan of action.

The growing disease burden of high BMI-related hepatitis B requires urgent attention. In this study, we found that death and DALY rates of hepatitis B attributable to a high BMI were significantly increased at a global level and in countries with high, middle, low-middle, and low SDI quintiles. This observation reflects the effect of overweight or obesity on the progression of liver diseases in patients with CHB. A prospective cohort study showed that coincidental central obesity doubled the risk of liver fibrosis progression in patients with CHB, and this association was independent of viral load and hepatitis activity [23]. An increase in BMI hindered the regression of fibrosis in patients with CHB on nucleoside analogue therapy in Asian [24] and European [25] cohorts. Emerging epidemiological evidence in addition to our global estimation indicates that overweight and obesity have a synergistic effect on inducing liver injury, fibrosis, or even the development of hepatocellular carcinoma in patients with CHB [26]. Therefore, lifestyle interventions should be emphasized for patients with CHB and an elevated BMI to halt disease progression and reduce the future disease burden.

Our findings of the burden of hepatitis B attributable to modifiable risk factors between regions and countries suggest the priority of taking action in the future. In 2019, countries with low, low-middle, and middle SDI quintiles had a high disease burden of hepatitis B attributable to modifiable risk factors. From 1990 to 2019, the age-standardized rate of hepatitis B caused by alcohol use was significantly increased in countries with a low-middle SDI, and the burden caused by a high BMI was increased globally and in countries with high, middle, low-middle, and low SDIs. The findings have two major implications for future implementation of strategies. Countries with low and middle socioeconomic development should prioritize addressing the high disease burden and the limited ability for intervention practice. Countries with a high SDI face the challenge of a high BMI causing hepatitis B.

This study has several strengths and limitations. A strength is that we examined the associations of viral infection with modifiable risk factors and comprehensively evaluated the disease burden of hepatitis B due to modifiable risk factors. This estimation could help lead to the integration of lifestyle interventions into plans for preventing hepatitis to achieve the goal of its elimination. However, a limitation is that the modifiable risk factors were restricted to tobacco, alcohol use, and a high BMI, whereas metabolic disorders, such as diabetes and dyslipidemia, have not been well studied [27–32]. Further studies should be conducted based on newly published epidemiological evidence.

## Conclusions

In conclusion, more than one-third of the disease burden of hepatitis B is attributable to tobacco, alcohol use, and a high BMI, and this contribution has increased from 1990 to 2019. This finding suggests the need for integration of lifestyle interventions into strategies for preventing hepatitis. Countries with low and middle socioeconomic development should prioritize focusing on the hepatitis B burden attributable to modifiable risk factors, and countries with a high SDI should be aware of the novel challenge of hepatitis B attributable to a high BMI.

## Electronic supplementary material

Below is the link to the electronic supplementary material.

Supplementary Material 1

## Data Availability

The datasets generated and/or analyzed during the current study are available in the Global Health Data Exchange (http://ghdx.healthdata.org).

## List of Abbreviations

AAPC: Average annual percentage change
BMI: Body mass index
CHB: Chronic hepatitis B virus infection
CI: Confidential interval
DALY: Disability-adjusted life-years
GBD: Global Burden of Diseases, Injuries, and Risk Factors Study
HBV: Hepatitis B virus
SDI: Socio-demographic index
UI: Uncertainty interval
WHO: World Health Organization
