# Supplementary material for: Global burden of hepatitis B attributable to modifiable risk factors from 1990 to 2019: a growing contribution and its association with socioeconomic status
Source: Global Health. 2023 Mar 31;19:23. doi: 10.1186/s12992-023-00922-z (PMC10064596; doi:10.1186/s12992-023-00922-z)
Supplement: Supplementary file 1 — Supplementary Material 1 [file 12992_2023_922_MOESM1_ESM.docx]

**Global burden of hepatitis B attributable to modifiable risk factors from 1990 to 2019: a growing contribution and its association with socioeconomic status**

**Supplemental Material**

**Supplemental Table 1.** All-age and age-standardized HBV related deaths and DALYs attributable to modifiable risk factors for both sexes combined in 2019 and average annual percentage change from 1990 to 2019, by GBD regions.

**Supplemental Table 2.** All-age and age-standardized HBV related deaths and DALYs attributable to modifiable risk factors for both sexes combined in 2019 and average annual percentage change from 1990 to 2019, by SDI quintiles.

**Supplemental Figure 1.** Hepatitis B related disease burden attributable to **modifiable risk factors**.

**Supplemental Figure 2.** Estimates of age-specific rates of risk-attributable HBV related deaths and DALYs, by SDI quintiles, 2019.

Supplemental Table 1. All-age and age-standardized HBV related deaths and DALYs attributable to modifiable risk factors for both sexes combined in 2019 and average annual percentage change from 1990 to 2019, by GBD regions.

|  | **Death** | | | | |  | **DALY** | | | | |
| --- | --- | --- | --- | --- | --- | --- | --- | --- | --- | --- | --- |
|  | **2019 number  No. (95% UI)** | **2019 ASR per 100,000 No. (95% UI)** | **1990 number  No. (95% UI)** | **1990 ASR per 100,000 No. (95% UI)** | **1990-2019 AAPC (95% CI)** |  | **2019 number  No. (95% UI)** | **2019 ASR per 100,000 No. (95% UI)** | **1990 number  No. (95% UI)** | **1990 ASR per 100,000 No. (95% UI)** | **1990-2019 AAPC (95% CI)** |
| **Tobacco** | | | | | | | | | | | |
| East Asia | 26487.80 (14244.71,40003.95) | 1.22 (0.66,1.83) | 30402.06 (15655.63,46630.82) | 3.21 (1.65,4.85) | -3.30 (-3.66,-2.94)* |  | 761943.34 (389710.74,1165120.76) | 34.69 (17.64,53.33) | 949572.19 (435678.51,1511447.22) | 95.08 (45.59,148.90) | -3.43 (-3.82,-3.04)* |
| Southeast Asia | 2861.82 (1303.04,4801.70) | 0.46 (0.22,0.76) | 1521.41 (715.95,2401.89) | 0.57 (0.29,0.90) | -0.79 (-1.12,-0.46)* |  | 79361.81 (33055.36,138405.59) | 11.80 (5.20,20.05) | 45783.81 (19143.02,73730.57) | 15.73 (7.17,25.21) | -1.04 (-1.37,-0.72)* |
| Oceania | 18.09 (6.11,32.10) | 0.25 (0.09,0.44) | 10.64 (4.10,17.82) | 0.35 (0.15,0.57) | -1.10 (-1.17,-1.03)* |  | 527.74 (149.43,950.15) | 6.55 (2.20,11.60) | 312.68 (108.07,533.20) | 9.19 (3.51,15.31) | -1.17 (-1.28,-1.07)* |
| Central Asia | 254.33 (116.66,435.45) | 0.32 (0.15,0.54) | 75.30 (34.91,129.65) | 0.16 (0.07,0.27) | 2.44 (2.21,2.66)* |  | 7661.7 (3124.97,13376.52) | 8.80 (3.82,15.02) | 2215.60 (957.30,3813.75) | 4.45 (1.95,7.67) | 2.33 (2.11,2.55)* |
| Central Europe | 313.77 (168.82,521.61) | 0.15 (0.08,0.25) | 435.37 (242.49,683.55) | 0.29 (0.16,0.45) | -2.20 (-2.37,-2.03)* |  | 7894.46 (4121.82,13198.78) | 4.08 (2.13,6.68) | 11872.22 (6344.70,18456.72) | 7.88 (4.14,12.24) | -2.28 (-2.45,-2.11)* |
| Eastern Europe | 456.42 (250.19,686.05) | 0.14 (0.07,0.20) | 206.88 (113.35,305.23) | 0.07 (0.04,0.11) | 2.28 (1.58,2.98)* |  | 12965.21 (6803.21,19742.74) | 4.06 (2.07,6.15) | 6084.14 (3149.18,8977.54) | 2.14 (1.11,3.17) | 2.27 (1.52,3.03)* |
| High-income Asia Pacific | 2472.7 (1388.92,3581.54) | 0.66 (0.36,0.96) | 1392.78 (817.39,1968.25) | 0.66 (0.39,0.94) | 0.05 (-0.23,0.32) |  | 61851.68 (33360.91,90265.76) | 18.65 (9.87,27.90) | 42342.24 (24179.26,59975.82) | 20 (11.41,28.35) | -0.20 (-0.49,0.08) |
| Australasia | 38.57 (18.36,66.00) | 0.09 (0.04,0.15) | 15.59 (7.94,25.53) | 0.07 (0.03,0.11) | 0.80 (0.46,1.15)* |  | 1046.57 (477.97,1802.30) | 2.47 (1.09,4.30) | 438.84 (221.40,714.25) | 1.95 (0.99,3.18) | 0.80 (0.49,1.11)* |
| Western Europe | 911.40 (489.02,1486.46) | 0.11 (0.06,0.18) | 585.86 (326.92,930.37) | 0.11 (0.06,0.17) | 0.15 (0.07,0.23)* |  | 21644.53 (11427.50,35182.28) | 2.89 (1.49,4.68) | 15085.09 (8154.79,23923.48) | 2.86 (1.52,4.48) | 0.02 (-0.05,0.10) |
| Southern Latin America | 62.75 (29.05,110.89) | 0.08 (0.04,0.13) | 30.01 (13.98,51.91) | 0.06 (0.03,0.11) | 0.61 (0.38,0.84)* |  | 1547.83 (686.80,2735.45) | 1.91 (0.84,3.40) | 802.78 (352.57,1424.27) | 1.70 (0.75,3.00) | 0.42 (0.20,0.63)* |
| High-income North America | 594.12 (331.99,930.58) | 0.10 (0.06,0.15) | 217.41 (130.21,320.24) | 0.06 (0.04,0.09) | 1.50 (1.37,1.63)* |  | 14961.32 (8365.85,23064.95) | 2.62 (1.48,4.05) | 5750.06 (3410.41,8306.11) | 1.78 (1.06,2.59) | 1.38 (1.26,1.50)* |
| Caribbean | 62.57 (29.99,107.23) | 0.12 (0.06,0.21) | 72.05 (36.17,120.14) | 0.28 (0.14,0.46) | -2.71 (-3.34,-2.09)* |  | 1541.63 (684.28,2709.79) | 2.96 (1.32,5.21) | 1852.31 (865.33,3091.81) | 7.01 (3.31,11.73) | -2.85 (-3.43,-2.27)* |
| Andean Latin America | 57.72 (20.13,108.54) | 0.11 (0.04,0.20) | 37.69 (12.60,70.75) | 0.19 (0.06,0.36) | -1.98 (-2.31,-1.64)* |  | 1293.19 (373.5,2459.29) | 2.31 (0.69,4.39) | 927.53 (258.50,1793.61) | 4.46 (1.38,8.55) | -2.15 (-2.50,-1.81)* |
| Central Latin America | 112.35 (38.32,214.22) | 0.05 (0.02,0.09) | 69.47 (25.67,125.79) | 0.08 (0.03,0.15) | -1.90 (-2.23,-1.56)* |  | 2787.14 (809.97,5353.35) | 1.15 (0.35,2.22) | 1915.02 (613.01,3506.26) | 2.14 (0.71,3.92) | -2.04 (-2.27,-1.80)* |
| Tropical Latin America | 166.73 (83.11,262.59) | 0.07 (0.03,0.11) | 87.11 (46.67,130.40) | 0.09 (0.05,0.14) | -1.03 (-1.20,-0.86)* |  | 4251.32 (2073.44,6659.80) | 1.70 (0.83,2.65) | 2521.74 (1303.26,3804.55) | 2.49 (1.31,3.78) | -1.31 (-1.49,-1.13)* |
| North Africa and Middle East | 1184.00 (631.72,1889.17) | 0.27 (0.14,0.42) | 547.29 (304.16,846.58) | 0.31 (0.17,0.48) | -0.54 (-0.71,-0.37)* |  | 33827.60 (17229.26,54238.82) | 6.96 (3.7,11.13) | 15766.62 (8491.33,24543.06) | 8.26 (4.50,12.74) | -0.60 (-0.87,-0.34)* |
| South Asia | 1391.85 (574.19,2361.77) | 0.10 (0.04,0.17) | 810.68 (339.43,1328.03) | 0.14 (0.06,0.23) | -1.23 (-1.35,-1.11)* |  | 36304.46 (13005.50,62684.16) | 2.45 (0.92,4.18) | 22961.16 (8485.82,38502.79) | 3.64 (1.49,6.01) | -1.35 (-1.47,-1.24)* |
| Central Sub-Saharan Africa | 18.87 (4.98,40.56) | 0.03 (0.01,0.07) | 11.76 (3.03,24.20) | 0.05 (0.01,0.10) | -1.37 (-1.55,-1.20)* |  | 557.31 (122.96,1206.36) | 0.91 (0.24,1.95) | 337.48 (79.64,685.64) | 1.31 (0.34,2.65) | -1.29 (-1.47,-1.11)* |
| Eastern Sub-Saharan Africa | 99.92 (20.52,200.29) | 0.06 (0.01,0.12) | 49.61 (9.39,101.29) | 0.07 (0.01,0.14) | -0.26 (-0.49,-0.04)* |  | 2769.44 (487.57,5507.88) | 1.56 (0.31,3.13) | 1363.00 (235.98,2795.66) | 1.68 (0.31,3.45) | -0.28 (-0.47,-0.08)* |
| Southern Sub-Saharan Africa | 165.05 (63.49,275.23) | 0.28 (0.11,0.45) | 117.15 (41.33,262.14) | 0.41 (0.15,0.90) | -1.21 (-1.58,-0.83)* |  | 5020.49 (1698.42,8591.58) | 7.88 (2.86,13.28) | 3598.96 (1129.42,8263.10) | 11.68 (3.79,26.63) | -1.33 (-1.60,-1.06)* |
| Western Sub-Saharan Africa | 337.70 (105.87,626.06) | 0.18 (0.06,0.33) | 190.60 (55.97,342.71) | 0.22 (0.07,0.39) | -0.61 (-0.74,-0.49)* |  | 9480.70 (2675.77,17679.99) | 4.53 (1.40,8.44) | 5248.84 (1280.90,9644.34) | 5.53 (1.51,10.05) | -0.67 (-0.83,-0.51)* |
| **Alcohol use** | | | | | | | | | | | |
| East Asia | 30719.37 (19161.80,44332.53) | 1.49 (0.93,2.14) | 38507.87 (25051.72,54853.83) | 3.92 (2.56,5.63) | -3.33 (-3.54,-3.13)* |  | 997648.45 (622449.18,1436253.29) | 48.25 (30.15,68.81) | 1394772.74 (895119.43,1969491.62) | 131.21 (84.56,185.51) | -3.45 (-3.59,-3.30)* |
| Southeast Asia | 13909.86 (8593.57,19975.00) | 2.03 (1.26,2.93) | 4628.93 (3025.2,6362.34) | 1.46 (0.94,2.03) | 1.14 (0.83,1.44)* |  | 509554.31 (310841.86,737023.42) | 70.18 (43.01,100.89) | 183370.43 (122698.16,252245.21) | 53.07 (34.81,72.97) | 1.03 (0.78,1.29)* |
| Oceania | 65.84 (23.07,134.44) | 0.63 (0.23,1.27) | 55.34 (25.51,93.06) | 1.27 (0.55,2.18) | -2.41 (-2.53,-2.28)* |  | 2889.10 (1014.13,5787.99) | 25.50 (9.13,51.64) | 2430.20 (1209.59,3984.31) | 51.59 (23.98,86.79) | -2.41 (-2.53,-2.28)* |
| Central Asia | 1773.93 (651.21,3174.37) | 1.93 (0.69,3.44) | 966.77 (436.59,1662.34) | 1.85 (0.81,3.28) | 0.17 (-0.38,0.72) |  | 72588.17 (29069.16,126111.58) | 75.45 (29.94,131.89) | 36767.77 (17927.45,59615.96) | 67.93 (32.10,112.52) | 0.42 (-0.16,1.01) |
| Central Europe | 3512.00 (1874.82,5379.74) | 1.95 (1.05,2.96) | 5607.59 (3513.69,7785.55) | 3.84 (2.43,5.31) | -2.34 (-2.64,-2.03)* |  | 110726.38 (60644.42,169234.92) | 66.28 (36.53,101.61) | 187030.16 (121195.49,256265.71) | 130.51 (85.12,178.65) | -2.35 (-2.65,-2.05)* |
| Eastern Europe | 5710.26 (2871.61,8530.46) | 2.02 (1.05,3.02) | 3001.88 (1621.95,4462.96) | 1.10 (0.61,1.62) | 2.24 (1.07,3.43)* |  | 226834.90 (120229.91,334207.89) | 84.45 (46.19,122.92) | 107063.34 (61364.62,153774.02) | 40.30 (23.50,57.40) | 2.71 (1.49,3.95)* |
| High-income Asia Pacific | 4328.16 (2583.39,6203.06) | 1.21 (0.74,1.70) | 7972.79 (5528.08,10382.94) | 3.91 (2.70,5.08) | -3.97 (-4.10,-3.84)* |  | 115167.43 (70260.51,159884.24) | 38.52 (23.86,53.11) | 277680.77 (196132.70,354840.42) | 135.32 (95.41,172.88) | -4.25 (-4.39,-4.12)* |
| Australasia | 312.47 (214.32,426.39) | 0.72 (0.50,0.98) | 274.02 (199.09,365.96) | 1.22 (0.89,1.63) | -1.80 (-2.12,-1.48)* |  | 8871.18 (6081.26,12014.55) | 22.41 (15.19,30.41) | 8486.25 (6129.13,11221.18) | 38.29 (27.47,50.50) | -1.80 (-2.11,-1.50)* |
| Western Europe | 3861.77 (2137.55,5755.40) | 0.49 (0.28,0.73) | 7263.26 (4509.30,10175.69) | 1.39 (0.88,1.93) | -3.54 (-3.70,-3.39)* |  | 98207.39 (54321.05,146137.84) | 14.45 (7.91,21.48) | 210219.51 (132916.08,293637.86) | 43.09 (26.71,60.19) | -3.74 (-3.90,-3.58)* |
| Southern Latin America | 953.18 (564.55,1449.32) | 1.18 (0.70,1.78) | 1261.21 (810.05,1814.84) | 2.73 (1.76,3.90) | -2.73 (-3.02,-2.43)* |  | 27071.61 (16492.01,40924.81) | 34.58 (21.01,52.36) | 39924.10 (24842.04,57154.64) | 85.42 (53.24,122.15) | -3.05 (-3.61,-2.48)* |
| High-income North America | 1285.91 (810.33,1801.30) | 0.23 (0.15,0.32) | 1037.92 (639.96,1467.68) | 0.32 (0.20,0.46) | -1.16 (-1.54,-0.78)* |  | 37384.00 (24101.16,51368.60) | 7.43 (4.88,10.10) | 35252.21 (22232.86,48965.90) | 11.30 (7.16,15.64) | -1.48 (-1.85,-1.11)* |
| Caribbean | 351.42 (124.04,661.01) | 0.68 (0.25,1.27) | 318.11 (130.57,543.95) | 1.15 (0.46,1.98) | -1.76 (-1.98,-1.53)* |  | 12367.63 (4542.60,22533.51) | 24.11 (9.00,43.94) | 11747.86 (4927.45,19863.39) | 41.16 (16.83,70.79) | -1.80 (-2.02,-1.57)* |
| Andean Latin America | 281.22 (70.89,577.38) | 0.47 (0.11,0.97) | 242.81 (73.48,448.80) | 0.95 (0.27,1.84) | -2.37 (-2.60,-2.14)* |  | 10778.11 (3284.25,21033.93) | 17.48 (5.26,34.66) | 10036.69 (3483.08,17828.74) | 37.21 (11.69,66.93) | -2.55 (-2.76,-2.34)* |
| Central Latin America | 1280.17 (658.32,1961.36) | 0.51 (0.26,0.79) | 1055.61 (626.89,1487.82) | 1.03 (0.59,1.49) | -2.45 (-2.83,-2.07)* |  | 46005.08 (24883.69,69322.79) | 18.00 (9.66,27.20) | 41928.41 (25858.34,57380.55) | 37.51 (22.28,51.94) | -2.57 (-2.90,-2.25)* |
| Tropical Latin America | 1619.17 (407.16,3048.70) | 0.64 (0.16,1.20) | 2009.33 (857.28,3336.16) | 1.71 (0.68,2.94) | -3.39 (-3.82,-2.96)* |  | 61216.84 (18333.63,108092.29) | 23.86 (7.19,42.52) | 86500.72 (40539.88,137751.10) | 69.11 (30.50,112.15) | -3.64 (-4.05,-3.23)* |
| North Africa and Middle East | 1516.28 (539.44,3165.26) | 0.30 (0.11,0.65) | 1596.53 (677.41,2822.00) | 0.81 (0.33,1.45) | -3.30 (-3.71,-2.89)* |  | 51853.80 (19785.90,104629.65) | 9.62 (3.55,19.92) | 55719.61 (24689.31,95882.67) | 26.08 (11.24,45.47) | -3.38 (-3.70,-3.05)* |
| South Asia | 30722.94 (12274.96,54362.33) | 1.87 (0.71,3.41) | 9880.62 (3003.34,20023.19) | 1.25 (0.34,2.63) | 1.54 (0.80,2.28)* |  | 1245083.66 (557114.58,2105960.60) | 72.16 (30.71,123.23) | 428793.03 (147204.80,829381.47) | 50.77 (16.14,101.30) | 1.36 (0.73,1.99)* |
| Central Sub-Saharan Africa | 2115.28 (1050.21,3648.58) | 3.16 (1.59,5.41) | 1272.61 (527.37,2196.02) | 4.73 (1.90,8.27) | -1.48 (-1.81,-1.14)* |  | 80604.17 (40261.92,140454.33) | 102.53 (50.67,176.67) | 46685.80 (20457.35,77530.98) | 150.95 (62.88,256.97) | -1.42 (-1.71,-1.11)* |
| Eastern Sub-Saharan Africa | 5180.83 (2769.98,8039.18) | 2.61 (1.35,4.16) | 3602.73 (2039.81,5644.16) | 4.20 (2.30,6.63) | -1.65 (-1.78,-1.52)* |  | 191147.16 (105715.61,292418.79) | 82.09 (44.21,127.51) | 128188.81 (75923.65,198289.63) | 130.19 (74.65,204.81) | -1.61 (-1.72,-1.51)* |
| Southern Sub-Saharan Africa | 1105.09 (682.42,1546.47) | 1.71 (1.04,2.41) | 1291.53 (875.28,1852.85) | 4.00 (2.61,5.87) | -2.89 (-3.63,-2.15)* |  | 41062.79 (26194.88,56933.70) | 57.94 (36.79,80.83) | 49784.81 (34326.18,69537.14) | 138.77 (95.05,196.64) | -3.03 (-3.68,-2.38)* |
| Western Sub-Saharan Africa | 14679.10 (8070.02,22382.12) | 6.72 (3.57,10.23) | 8561.51 (4664.10,13348.07) | 8.76 (4.72,13.77) | -0.91 (-1.02,-0.81)* |  | 521293.15 (295971.92,789688.99) | 201.43 (111.81,306.47) | 292225.87 (161647.32,448533.88) | 266.20 (145.56,412.61) | -0.96 (-1.09,-0.83)* |
| **High BMI** | | | | | | | | | | | |
| East Asia | 13342.28 (4161.01,28651.45) | 0.63 (0.20,1.35) | 7523.81 (1411.47,19592.89) | 0.76 (0.14,1.99) | -0.69 (-0.95,-0.42)* |  | 426801.23 (134930.51,909907.66) | 20.16 (6.37,43.08) | 260206.29 (48626.42,682660.14) | 24.86 (4.64,65.25) | -0.72 (-0.99,-0.45)* |
| Southeast Asia | 2096.71 (756.88,4154.93) | 0.32 (0.12,0.64) | 429.80 (115.48,989.10) | 0.15 (0.04,0.33) | 2.72 (2.52,2.92)* |  | 66551.10 (24013.54,129117.59) | 9.47 (3.43,18.46) | 15177.18 (4199.50,35169.09) | 4.72 (1.29,10.90) | 2.41 (2.20,2.62)* |
| Oceania | 23.28 (9.29,41.65) | 0.29 (0.11,0.52) | 9.72 (3.86,18.72) | 0.28 (0.11,0.55) | 0.06 (-0.11,0.23) |  | 786.91 (319.91,1376.97) | 8.56 (3.43,15.32) | 334.79 (135.84,634.07) | 8.69 (3.48,16.75) | -0.08 (-0.23,0.07) |
| Central Asia | 332.40 (135.75,636.67) | 0.40 (0.16,0.75) | 63.25 (23.40,128.46) | 0.13 (0.05,0.26) | 3.94 (3.70,4.19)* |  | 10838.89 (4418.32,20100.27) | 12.10 (4.99,22.52) | 1969.49 (741.07,4002.97) | 3.89 (1.47,7.86) | 3.95 (3.66,4.23)* |
| Central Europe | 340.73 (138.80,618.51) | 0.17 (0.07,0.31) | 365.81 (152.95,665.27) | 0.25 (0.10,0.45) | -1.30 (-1.46,-1.15)* |  | 8779.42 (3577.20,15948.14) | 4.70 (1.92,8.53) | 10228.55 (4264.52,18810.78) | 6.87 (2.90,12.62) | -1.34 (-1.49,-1.19)* |
| Eastern Europe | 490.48 (217.12,874.28) | 0.15 (0.07,0.26) | 181.29 (73.52,328.86) | 0.06 (0.03,0.12) | 2.99 (2.35,3.64)* |  | 14136.68 (6111.36,24970.98) | 4.56 (1.97,8.11) | 5364.42 (2183.30,9709.55) | 1.91 (0.77,3.47) | 3.06 (2.36,3.76)* |
| High-income Asia Pacific | 1234.93 (384.74,2676.72) | 0.35 (0.11,0.75) | 411.92 (102.90,963.64) | 0.20 (0.05,0.46) | 1.98 (1.75,2.21)* |  | 32492.46 (9881.23,70787.31) | 10.28 (3.19,22.46) | 13192.81 (3373.91,31004.75) | 6.28 (1.60,14.72) | 1.73 (1.50,1.95)* |
| Australasia | 61.77 (24.68,114.48) | 0.14 (0.06,0.26) | 13.20 (5.02,25.52) | 0.06 (0.02,0.11) | 3.04 (2.87,3.20)* |  | 1688.29 (683.02,3083.77) | 4.11 (1.69,7.39) | 395.95 (151.67,757.15) | 1.77 (0.68,3.39) | 2.91 (2.74,3.09)* |
| Western Europe | 828.78 (325.50,1561.10) | 0.10 (0.04,0.20) | 380.33 (140.67,747.27) | 0.07 (0.03,0.14) | 1.35 (1.27,1.43)* |  | 20395.77 (8140.92,38960.18) | 2.86 (1.16,5.47) | 10175.27 (3831.21,20077.27) | 1.97 (0.74,3.91) | 1.27 (1.19,1.36)* |
| Southern Latin America | 65.11 (24.58,125.42) | 0.08 (0.03,0.15) | 18.97 (6.50,38.48) | 0.04 (0.01,0.08) | 2.35 (2.14,2.56)* |  | 1688.03 (648.48,3253.98) | 2.11 (0.82,4.09) | 536.12 (182.31,1114.87) | 1.14 (0.39,2.36) | 2.20 (1.99,2.41)* |
| High-income North America | 771.74 (330.76,1359.97) | 0.13 (0.06,0.23) | 167.69 (69.18,305.51) | 0.05 (0.02,0.09) | 3.42 (3.30,3.53)* |  | 21111.77 (9043.39,37364.27) | 3.93 (1.71,6.91) | 4919.39 (2047.04,8885.99) | 1.54 (0.64,2.81) | 3.28 (3.17,3.39)* |
| Caribbean | 78.16 (29.96,151.35) | 0.15 (0.06,0.29) | 60.42 (22.89,115.83) | 0.23 (0.09,0.44) | -1.32 (-1.82,-0.81)* |  | 2296.69 (882.10,4389.91) | 4.45 (1.71,8.48) | 1803.64 (683.02,3479.76) | 6.58 (2.47,12.67) | -1.36 (-1.77,-0.95)* |
| Andean Latin America | 156.52 (60.83,284.21) | 0.28 (0.11,0.50) | 65.69 (24.46,124.84) | 0.31 (0.11,0.59) | -0.29 (-0.63,0.05) |  | 4275.78 (1690.42,7724.29) | 7.34 (2.89,13.22) | 1973.19 (751.24,3701.11) | 8.57 (3.25,15.92) | -0.50 (-0.82,-0.17)* |
| Central Latin America | 221.46 (90.71,407.28) | 0.09 (0.04,0.17) | 69.28 (24.31,137.75) | 0.08 (0.03,0.15) | 0.68 (0.38,0.99)* |  | 6402.80 (2597.39,11457.00) | 2.58 (1.06,4.64) | 2233.65 (801.18,4429.14) | 2.26 (0.81,4.47) | 0.53 (0.22,0.85)* |
| Tropical Latin America | 220.71 (93.04,393.64) | 0.09 (0.04,0.16) | 54.03 (20.21,106.56) | 0.05 (0.02,0.11) | 1.77 (1.58,1.95)* |  | 6478.79 (2740.51,11537.23) | 2.56 (1.08,4.57) | 1797.74 (662.89,3540.81) | 1.64 (0.62,3.23) | 1.56 (1.35,1.78)* |
| North Africa and Middle East | 1634.94 (654.15,2938.37) | 0.35 (0.14,0.63) | 484.98 (190.22,974.58) | 0.26 (0.10,0.52) | 1.01 (0.81,1.20)* |  | 50971.40 (20156.99,92229.25) | 9.95 (3.93,17.91) | 15340.05 (6070.62,30434.37) | 7.60 (3,15.21.00) | 0.93 (0.66,1.21)* |
| South Asia | 1133.46 (419.21,2269.47) | 0.08 (0.03,0.15) | 194.09 (48.00,499.62) | 0.03 (0.01,0.08) | 3.13 (2.80,3.45)* |  | 35728.52 (13075.14,71396.90) | 2.25 (0.82,4.49) | 6411.55 (1600.40,16510.39) | 0.91 (0.23,2.35) | 3.17 (2.95,3.38)* |
| Central Sub-Saharan Africa | 30.93 (10.26,65.56) | 0.05 (0.02,0.11) | 12.23 (3.84,26.62) | 0.05 (0.02,0.10) | 0.02 (-0.08,0.12) |  | 1076.70 (353.67,2286.20) | 1.50 (0.49,3.16) | 407.82 (125.75,903.00) | 1.42 (0.44,3.09) | 0.14 (0.04,0.25)* |
| Eastern Sub-Saharan Africa | 156.38 (54.05,321.45) | 0.08 (0.03,0.17) | 33.25 (8.24,83.97) | 0.04 (0.01,0.10) | 2.62 (2.53,2.71)* |  | 5392.20 (1959.81,11108.35) | 2.50 (0.86,5.12) | 1132.58 (275.81,2789.51) | 1.20 (0.29,2.99) | 2.58 (2.48,2.69)* |
| Southern Sub-Saharan Africa | 283.98 (121.08,507.87) | 0.45 (0.19,0.80) | 101.18 (36.10,229.27) | 0.32 (0.11,0.74) | 1.17 (0.74,1.59)* |  | 9878.55 (4137.64,17626.74) | 14.39 (6.09,25.69) | 3626.86 (1318.78,8077.23) | 10.62 (3.86,23.64) | 1.07 (0.70,1.44)* |
| Western Sub-Saharan Africa | 568.78 (220.58,1101.71) | 0.27 (0.11,0.54) | 156.95 (48.12,357.78) | 0.17 (0.05,0.38) | 1.78 (1.64,1.92)* |  | 18459.62 (7103.39,35554.36) | 7.79 (3.02,15.19) | 5029.27 (1569.71,11204.90) | 4.84 (1.50,10.88) | 1.67 (1.50,1.84)* |

*Changes that are statically significant.

AAPC, average annual percentage change; BMI, body mass index; CI, confidence interval; DALY, disability-adjusted life-years; UI, uncertainty interval.

Supplemental Table 2. All-age and age-standardized HBV related deaths and DALYs attributable to modifiable risk factors for both sexes combined in 2019 and average annual percentage change from 1990 to 2019, by SDI quintiles.

|  | **Death** | | | | |  | **DALY** | | | | |
| --- | --- | --- | --- | --- | --- | --- | --- | --- | --- | --- | --- |
|  | **2019 number  No. (95% UI)** | **2019 ASR per 100,000 No. (95% UI)** | **1990 number  No. (95% UI)** | **1990 ASR per 100,000 No. (95% UI)** | **1990-2019 AAPC (95% CI)** |  | **2019 number  No. (95% UI)** | **2019 ASR per 100,000 No. (95% UI)** | **1990 number  No. (95% UI)** | **1990 ASR per 100,000 No. (95% UI)** | **1990-2019 AAPC (95% CI)** |
| **Tobacco** | | | | | | | | | | | |
| Global | 38068.53 (20928.86,56370.11) | 0.46 (0.25,0.67) | 36886.71 (19386.53,55625.27) | 0.89 (0.47,1.33) | -2.30 (-2.56,-2.04)* |  | 1069239.47 (563985.53,1627720.20) | 12.65 (6.69,19.24) | 1136752.31 (533723.28,1764841.40) | 26.57 (12.84,40.97) | -2.56 (-2.79,-2.33)* |
| High SDI | 4286.11 (2452.22,6361.39) | 0.25 (0.14,0.37) | 2568.16 (1507.69,3688.28) | 0.26 (0.15,0.37) | -0.17 (-0.34,0.01) |  | 107560.13 (59817.92,159032.03) | 6.66 (3.57,9.85) | 75163.65 (42039.21,109201.37) | 7.79 (4.32,11.35) | -0.54 (-0.73,-0.35)* |
| High-middle SDI | 8871.20 (4765.68,13501.43) | 0.43 (0.23,0.66) | 11959.43 (6289.68,18243.47) | 1.08 (0.57,1.65) | -3.18 (-3.59,-2.77)* |  | 248724.57 (126702.12,386976.20) | 12.28 (6.24,19.04) | 361409.66 (172778.80,567219.25) | 32.31 (15.38,50.66) | -3.32 (-3.72,-2.92)* |
| Middle SDI | 20304.95 (10909.47,30212.68) | 0.78 (0.43,1.15) | 18867.89 (9661.08,28503.57) | 1.69 (0.88,2.53) | -2.63 (-2.99,-2.27)* |  | 582810.44 (298062.17,895363.55) | 21.4 (11.19,32.57) | 593491.24 (268150.48,928354.13) | 49.86 (24.23,76.67) | -2.85 (-3.26,-2.44)* |
| Low-middle SDI | 3977.56 (2119.60,6134.96) | 0.28 (0.15,0.44) | 3135.31 (1596.22,4828.28) | 0.49 (0.26,0.74) | -1.92 (-2.19,-1.64)* |  | 112543.72 (55601.14,176068.62) | 7.61 (3.92,11.89) | 96803.58 (44157.44,154052.18) | 13.97 (6.71,21.95) | -2.13 (-2.40,-1.86)* |
| Low SDI | 619.90 (211.74,1102.93) | 0.12 (0.05,0.21) | 350.01 (124.83,610.61) | 0.15 (0.06,0.26) | -0.76 (-0.86,-0.66)* |  | 17363.60 (5278.31,31536.48) | 3.06 (1.02,5.44) | 9719.84 (2908.07,17275.70) | 3.76 (1.27,6.56) | -0.71 (-0.81,-0.62)* |
| **Alcohol use** | | | | | | | | | | | |
| Global | 125284.25 (72232.01,183697.10) | 1.51 (0.87,2.22) | 100408.96 (63874.48,140599.40) | 2.34 (1.48,3.29) | -1.51 (-1.72,-1.30)* |  | 4468355.30 (2696885.00,6440347.57) | 53.64 (32.42,77.54) | 3634609.10 (2365360.62,5080844.32) | 80.67 (51.77,112.51) | -1.41 (-1.66,-1.15)* |
| High SDI | 10981.45 (6483.96,15716.53) | 0.67 (0.41,0.95) | 16741.58 (11269.67,22090.21) | 1.73 (1.17,2.29) | -3.19 (-3.35,-3.04)* |  | 308740.80 (186044.97,436411.64) | 21.24 (13.14,29.67) | 558908.09 (384157.34,735264.03) | 59.37 (40.88,78.40) | -3.48 (-3.64,-3.32)* |
| High-middle SDI | 24575.91 (14187.54,35681.89) | 1.26 (0.74,1.82) | 28528.86 (18364.06,39245.96) | 2.58 (1.66,3.52) | -2.52 (-2.95,-2.08)* |  | 844569.93 (511086.90,1200274.29) | 44.60 (26.96,62.94) | 987226.54 (652253.95,1347964.40) | 87.01 (57.14,119.15) | -2.36 (-2.86,-1.86)* |
| Middle SDI | 38763.04 (23286.03,55828.97) | 1.48 (0.88,2.12) | 31061.48 (20102.59,43785.22) | 2.57 (1.64,3.65) | -1.91 (-2.05,-1.77)* |  | 1366258.64 (845992.84,1919836.50) | 50.20 (31.01,70.17) | 1178690.43 (769190.51,1649085.33) | 89.09 (57.76,125.43) | -1.97 (-2.07,-1.87)* |
| Low-middle SDI | 35353.40 (17969.82,55562.37) | 2.28 (1.15,3.61) | 15289.27 (7968.05,24730.56) | 2.12 (1.10,3.44) | 0.24 (-0.01,0.50) |  | 1359882.00 (714524.44,2099305.52) | 83.13 (43.56,129.09) | 590501.83 (316374.49,931514.36) | 74.41 (39.34,118.94) | 0.38 (0.10,0.66)* |
| Low SDI | 15567.90 (8288.52,24663.43) | 2.48 (1.27,3.97) | 8757.84 (4505.32,14030.82) | 3.16 (1.57,5.12) | -0.84 (-0.93,-0.74)* |  | 587380.62 (321344.18,898261.48) | 82.21 (43.55,129.00) | 318194.34 (163754.80,504131.97) | 102.15 (52.66,163.32) | -0.75 (-0.86,-0.64)* |
| **High body mass index** | | | | | | | | | | | |
| Global | 24073.53 (8741.33,48325.58) | 0.29 (0.10,0.58) | 10797.90 (2694.77,26446.74) | 0.25 (0.06,0.62) | 0.43 (0.24,0.62)* |  | 746231.59 (264307.76,1470149.49) | 8.86 (3.15,17.47) | 362256.65 (88411.08,895554.92) | 8.23 (2.02,20.29) | 0.24 (0.05,0.43)* |
| High SDI | 3224.06 (1247.44,6156.00) | 0.19 (0.08,0.37) | 1155.06 (383.26,2456.56) | 0.12 (0.04,0.25) | 1.73 (1.58,1.87)* |  | 86589.54 (33946.34,163336.21) | 5.64 (2.21,10.74) | 35250.48 (11615.42,74621.34) | 3.68 (1.21,7.84) | 1.48 (1.32,1.64)* |
| High-middle SDI | 5884.43 (2104.04,11605.50) | 0.29 (0.10,0.58) | 3803.43 (990.78,9253.92) | 0.34 (0.09,0.83) | -0.59 (-1.10,-0.08)* |  | 181667.58 (64652.15,360900.34) | 9.25 (3.27,18.45) | 124728.33 (31244.26,304193.16) | 11.03 (2.77,26.87) | -0.67 (-1.22,-0.12)* |
| Middle SDI | 12038.04 (4136.06,24204.45) | 0.45 (0.16,0.92) | 4904.36 (1061.34,12365.92) | 0.42 (0.09,1.05) | 0.31 (-0.05,0.68) |  | 383739.98 (130833.96,772624.87) | 13.99 (4.78,28.18) | 170853.77 (36751.05,438508.98) | 13.52 (2.93,34.38) | 0.14 (-0.27,0.55) |
| Low-middle SDI | 2204.43 (792.07,4660.01) | 0.15 (0.05,0.32) | 722.16 (158.56,1852.82) | 0.11 (0.02,0.28) | 1.19 (1.03,1.36)* |  | 69999.39 (25215.38,145379.81) | 4.55 (1.64,9.48) | 24506.73 (5342.62,64507.23) | 3.34 (0.73,8.67) | 1.07 (0.90,1.24)* |
| Low SDI | 713.54 (263.68,1454.04) | 0.12 (0.05,0.25) | 209.10 (59.35,499.17) | 0.08 (0.02,0.19) | 1.44 (1.38,1.50)* |  | 23958.39 (8535.73,48768.42) | 3.70 (1.35,7.44) | 6798.65 (1925.47,15849.62) | 2.37 (0.68,5.57) | 1.56 (1.49,1.62)* |

*Changes that are statically significant.

AAPC, average annual percentage change; BMI, body mass index; CI, confidence interval; DALY, disability-adjusted life-years; SDI, Socio-Demographic Index; UI, uncertainty interval.


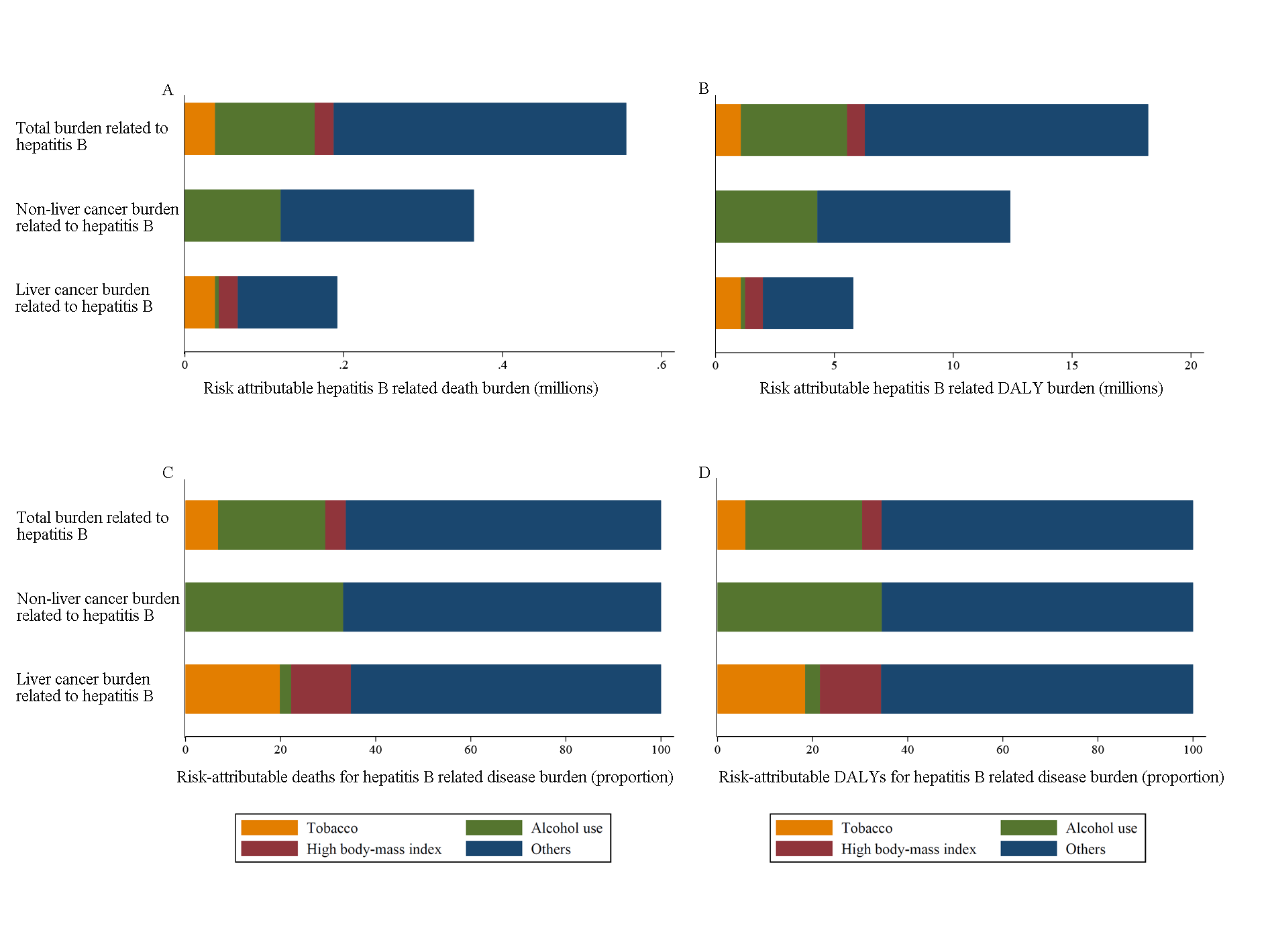


Supplemental Figure 1. Hepatitis B related disease burden attributable to modifiable risk factors. (A) Hepatitis B related death numbers attributable to risk factors in 2019; (B) Hepatitis B related DALYs attributable to risk factors in 2019; (C) Proportion of hepatitis B related deaths attributable to risk factors in 2019; (D) Proportion of hepatitis B related DALYs attributable to risk factors in 2019. DALY, disability-adjusted life-years.


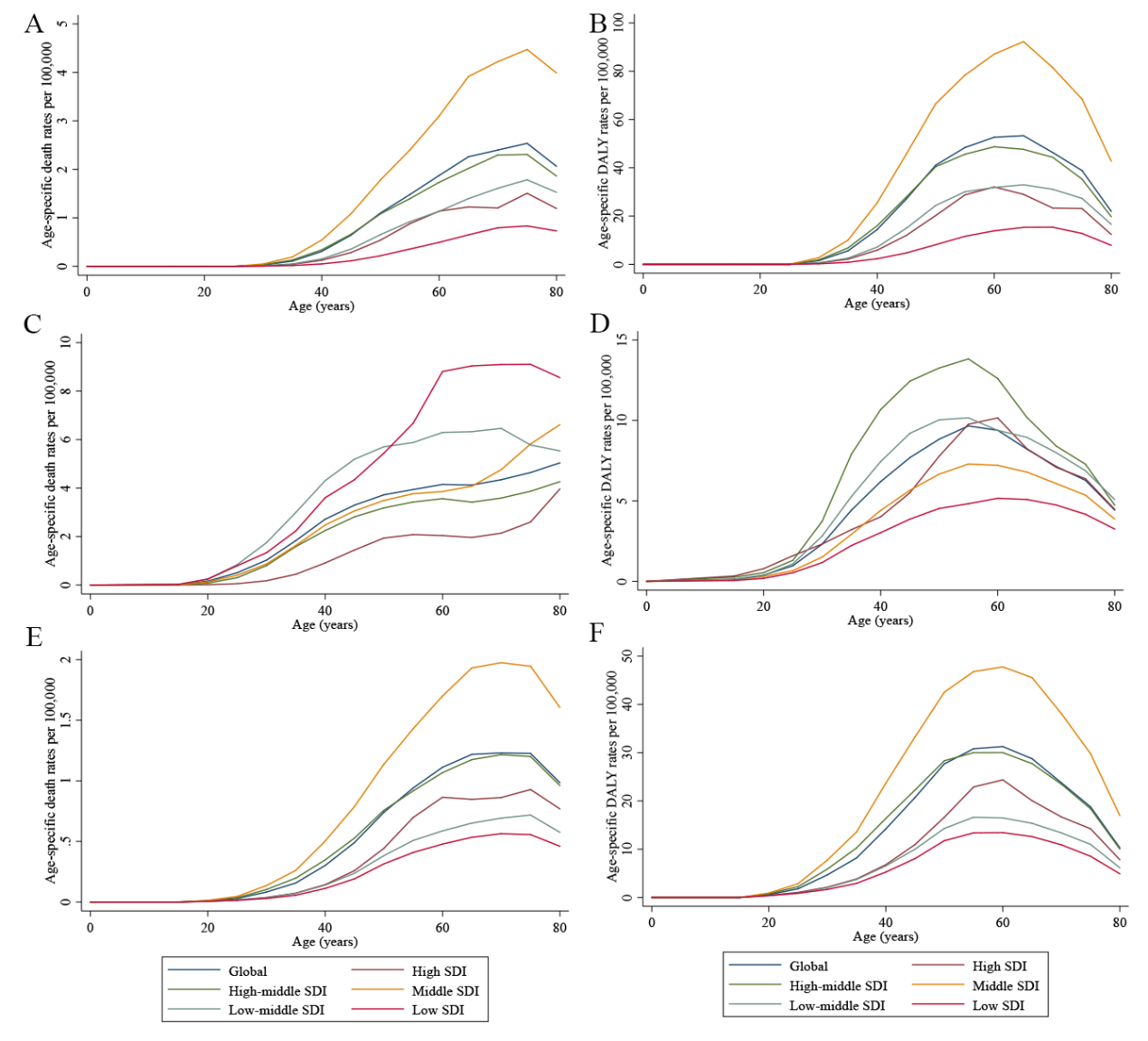


Supplemental Figure 2. Estimates of age-specific rates of risk-attributable HBV related deaths and DALYs, by SDI quintiles, 2019. (A) Tobacco attributable death rate; (B) Tobacco attributable DALY rate; (C) Alcohol use attributable death rate; (D) Alcohol use attributable DALY rate; (E) High BMI attributable death rate; (F) High BMI attributable DALY rate. BMI, body mass index; DALY, disability-adjusted life-years. BMI, body mass index; DALY, disability-adjusted life-years; SDI, Socio-Demographic Index.
